# Personal

**Published:** 1914-07

**Authors:** 


### PERSONAL.

---

Announcement of removal, travel, and other matters of interest are requested. Please report errors in the listing of any physician in the State and other directories, that we may co-operate with the State Society in securing a correct list.

---

Our Associate Editor, Sir William Osler, has been elected foreign associate of the Paris Academy of Medicine.

---

Dr. Bernard Bartow, of Buffalo, has removed his office and residence to 503 Delaware ave.

---

Dr. F. Park Lewis, of Buffalo, delivered the address to the graduating class of the State School for the Blind at Batavia, June 10.

---

Miss Maude E. Ragar has resigned the position of superintendent of the Brooks Memorial Hospital of Dunkirk.

---

Dr. and Mrs. Ray H. Johnson, of Buffalo, sail July 2 on the Baltic from New York, for a tour of Ireland, Scotland and

England, reaching London in time for the Clinical Congress of North American Surgeons, July 25 to Aug. 3.

---

Dr. Charles E. Congdon, of Buffalo, has returned from the Isle of Pines.

---

Dr. C. F. Smith, of Ransomville, was struck by an automobile in Buffalo, June 4, and was quite seriously injured, requiring treatment at the Emergency Hospital.

Dr. William G. Taylor, of Buffalo, has been appointed Assistant Obstetrician to the Buffalo General Hospital.

---

Dr. George J. Eckel, of Buffalo, has gone to Europe for several months.

---

Dr. and Mrs. W. W. Quinton, of Buffalo, have received a prize of $50 for a one-act play entitled "The Finger of Fate," which has been staged by the Bonstell Co. at the Star Theatre.

